# Supplementary material for: A Study of Catalytic Oxidation of a Library of C2 to C4 Alcohols in the Presence of Nanogold
Source: Nanomaterials (Basel). 2019 Mar 15;9(3):442. doi: 10.3390/nano9030442 (PMC6474078; doi:10.3390/nano9030442)
Supplement: Supplementary file 1 [file nanomaterials-09-00442-s001.pdf]

Supplementary Materials

A Study of Catalytic Oxidation of a Library of C_2 to C_4 Alcohols in the Presence of Nanogold

Maciej Kapkowski [1], Anna Niemczyk-Wojdyla [1], Piotr Bartczak [1], Monika Pyrkosz Bulska [1], Kamila Gajcy [1], Rafal Sitko [1], Maciej Zubko [2,3], Jacek Szade [4], Joanna Klimontko [4], Katarzyna Balin [4] and Jaroslaw Polanski [1,*]

[1] Institute of Chemistry, University of Silesia, Szkolna 9, 40-006 Katowice, Poland; maciej.kapkowski@us.edu.pl (M.K.); anna.niemczyk@us.edu.pl (A.N.-W.); piotr.bartczak@us.edu.pl (P.B.); monika.pyrkosz-bulska@us.edu.pl (M.P.B.); kamila.gajcy@us.edu.pl (K.G.); rafal.sitko@us.edu.pl (R.S.)

[2] Institute of Materials Science, University of Silesia, 75 Pułku Piechoty 1A, 41-500 Chorzów, Poland; maciej.zubko@us.edu.pl

[3] Department of Physics, University of Hradec Králové, 500-03 Rokitanského 62, Hradec Králové, Czech Republic

[4] Institute of Physics, University of Silesia, 75 Pułku Piechoty 1A, 41-500 Chorzów, Poland; jacek.szade@us.edu.pl (J.S.); joanna.klimontko@us.edu.pl (J.K.); katarzyna.balin@us.edu.pl (K.B.)

* Correspondence: jaroslaw.polanski@us.edu.pl; Tel.: +48-32-2599978

Table of contents

Table S1. The amount of the precursor (g) per 100 g of tetraethyl orthosilicate that was required for the syntheses of the catalysts in Experiment 2.1.1.

Figure S1. The ^{1}H NMR (**A**), ^{13}C NMR (**B**), COSY (**C**), HMQC (**D**) spectra of the crude reaction mixture for the catalytic oxidation of 2-propanol in the presence of 0.1% Au/SiO_2 under the reaction conditions as described in Experiment 2.3.

Figure S2. The ^{1}H NMR (**A**), ^{13}C NMR (**B**), COSY (**C**), HMQC (**D**) spectra of the crude reaction mixture for the oxidation of 1,3-propanediol in the presence of 0.1% Au/SiO_2 under the reaction conditions as described in Experiment 2.3.

Table 1. The amount of the precursor (g) per 100 g of tetraethyl orthosilicate that was required for the syntheses of the catalysts in Experiment 2.1.1.

Catalyst	Nanometal content [mmol]		Amount of the precursor [g]	
	Au	Pd	30% $HAuCl_4$	$PdCl_2$
0.1% Au/SiO_2	0.147	-	0.166	-
0.7% Au/SiO_2	1.032	-	1.169	-
(1.1% Pd; 0.4% Au)/ SiO_2	0.595	3.026	0.673	0.5366
(0.2% Pd; 1.1% Au)/SiO_2	1.632	0.549	1.848	0.0974

A

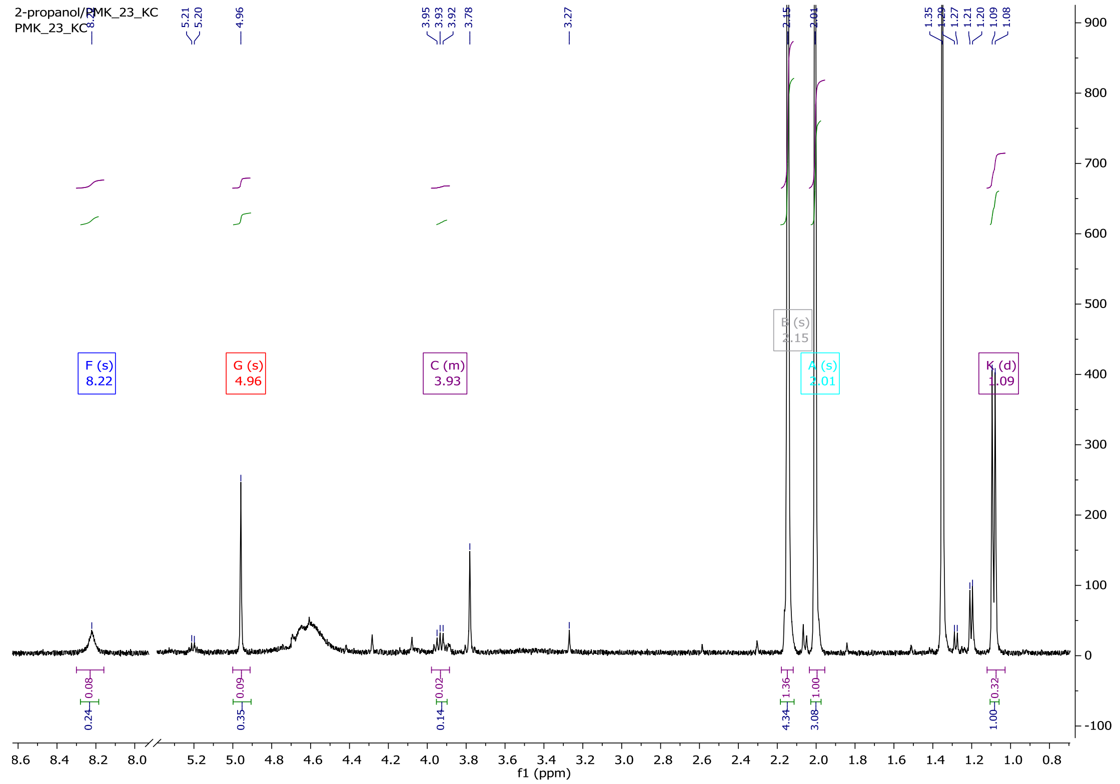

2-propanol/PMK_23_KC
PMK_23_KC
F (s) 8.22
G (s) 4.96
C (m) 3.93
A (s) 2.01
K (d) 1.09
f1 (ppm)

B

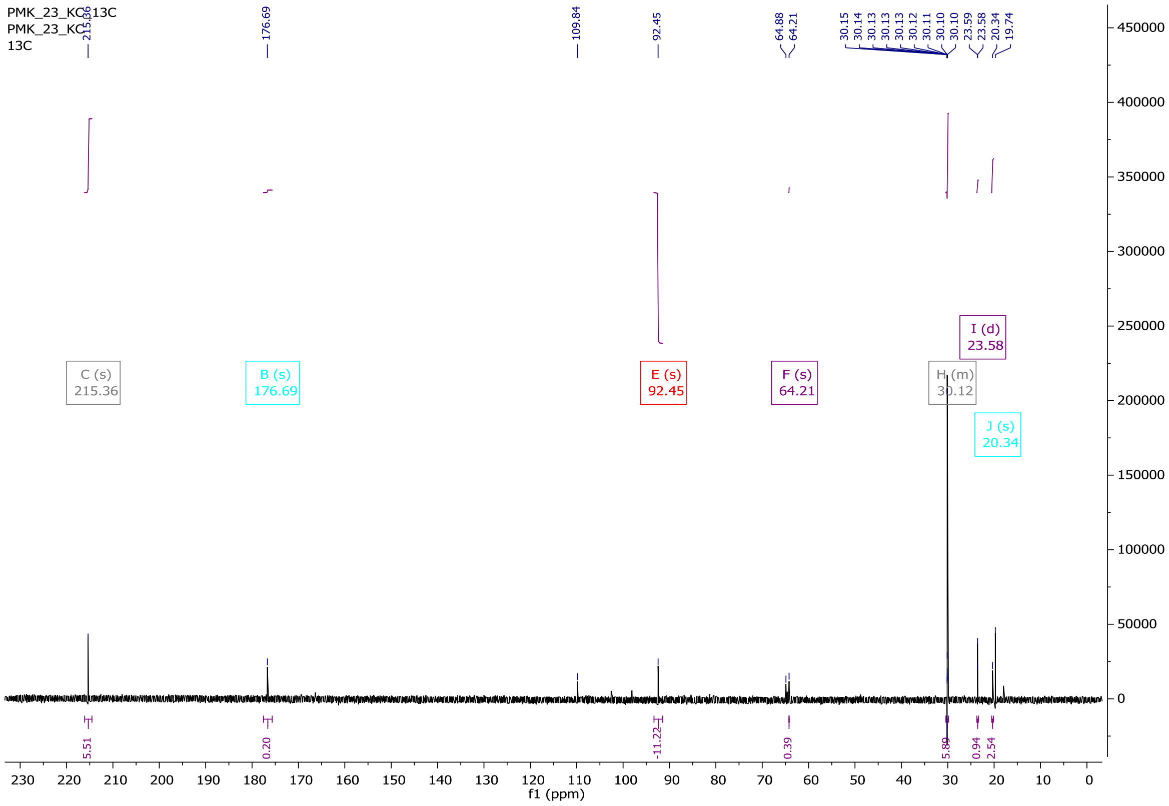

PMK_23_KC 13C
PMK_23_KC
13C
C (s) 215.36
B (s) 176.69
E (s) 92.45
F (s) 64.21
H (m) 30.12
I (d) 23.58
J (s) 20.34
f1 (ppm)

C

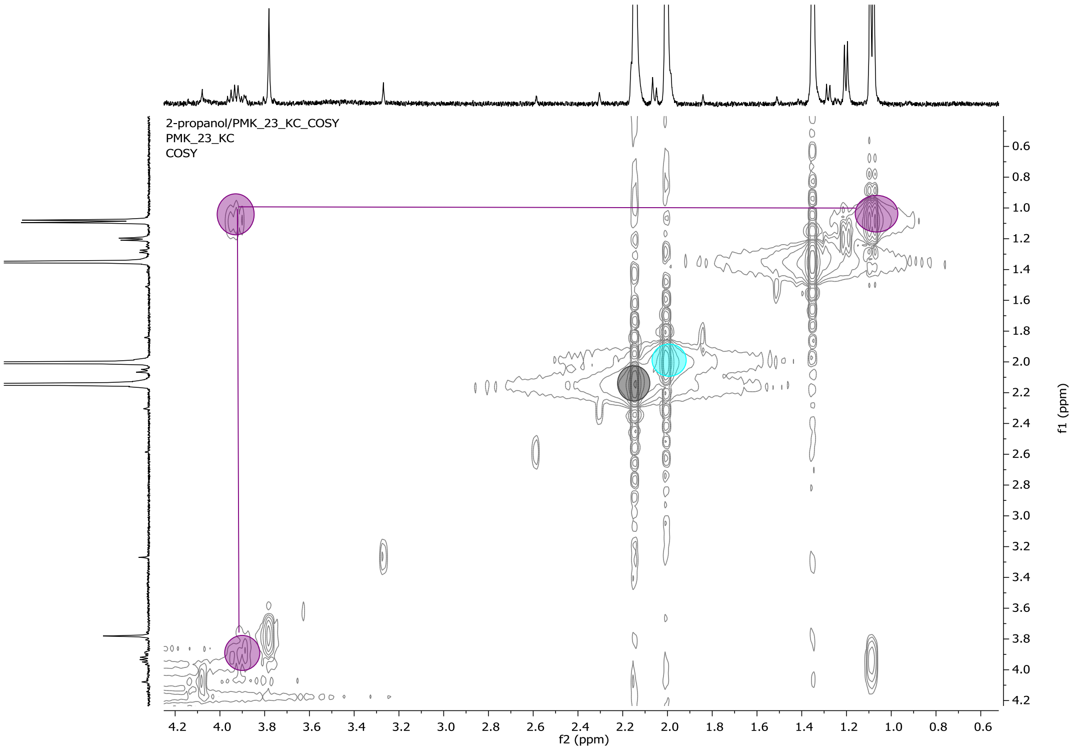

D

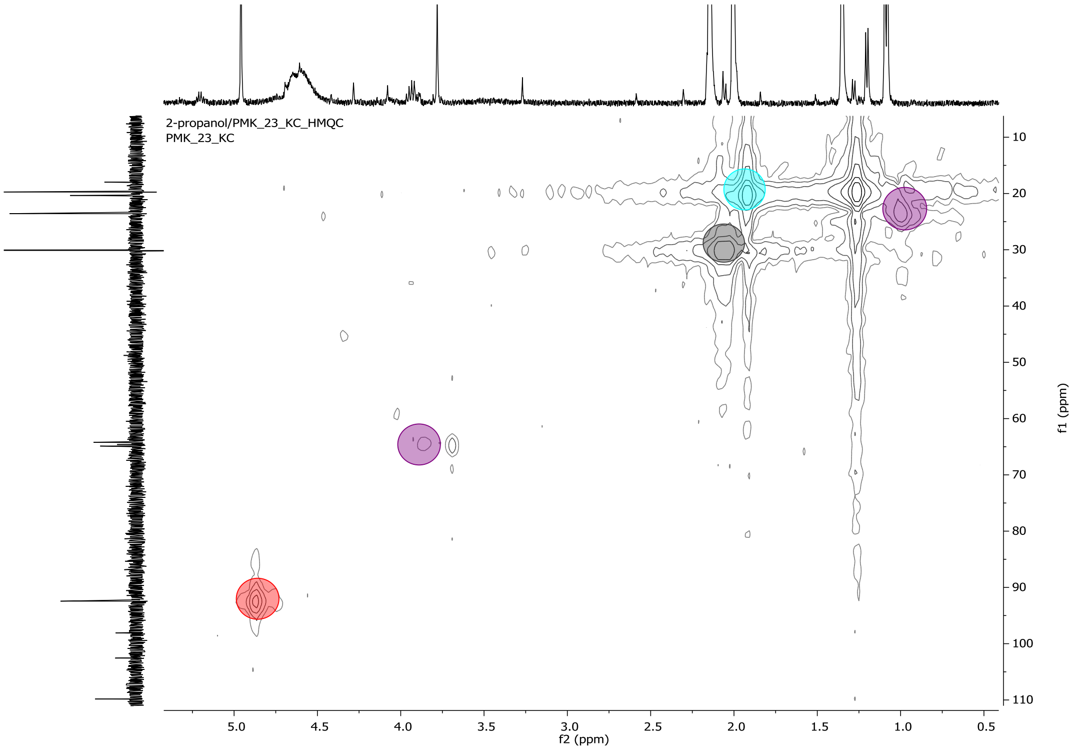

Figure 1. The ^{1}H NMR (**A**), ^{13}C NMR (**B**), COSY (**C**) and HMQC (**D**) spectra of the crude reaction mixture that was required for the oxidation of 2-propanol in the presence of 0.1% Au/SiO_2 under the reaction conditions as described in Experiment 2.3. Specific signals on the spectra are assigned to the products of the reaction, namely: ● 2-propanol, ● acetic acid, ● formic acid, ● acetone, ● 1,3,5-trioxane. Other products were not marked on the spectra. Measurements of the samples were taken in D_2O at Bruker 400 MHz.

A

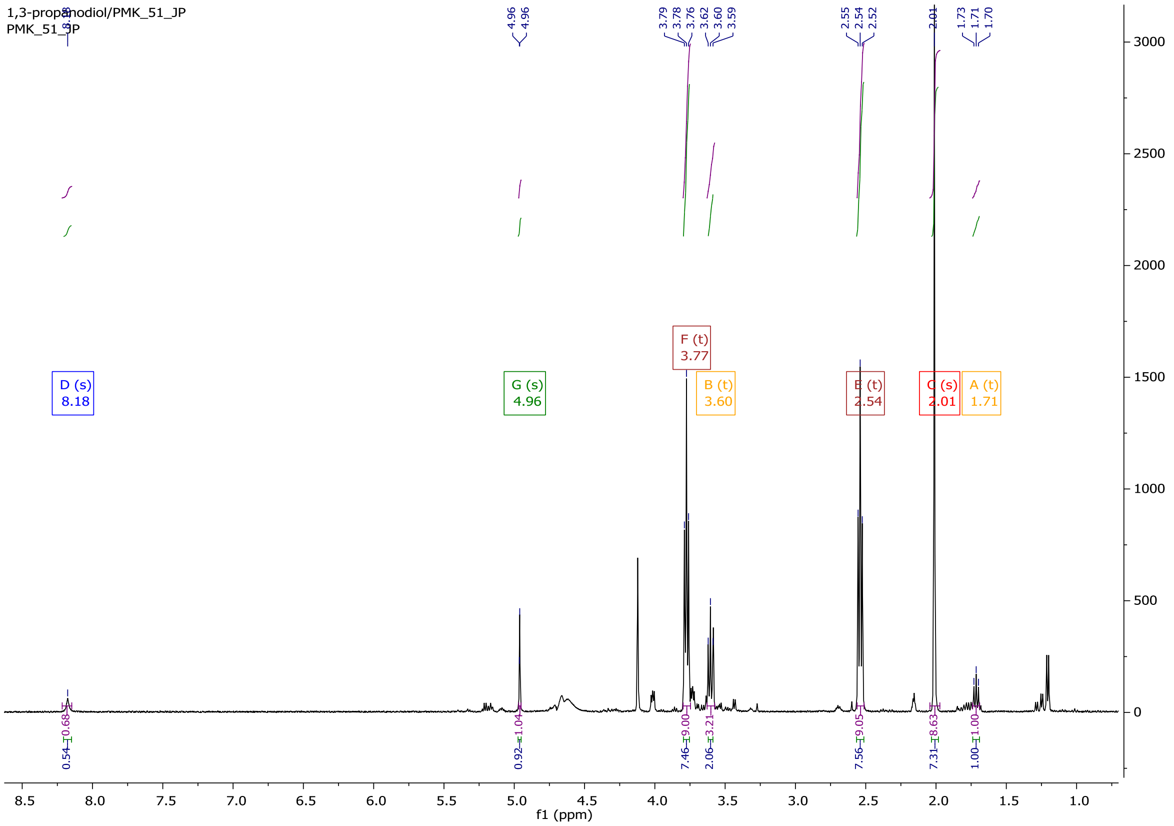
1,3-propanodiol/PMK_51_JP
PMK_51_JP
F (t) 3.77
D (s) 8.18
G (s) 4.96
B (t) 3.60
E (t) 2.54
C (s) 2.01
A (t) 1.71
f1 (ppm)

B

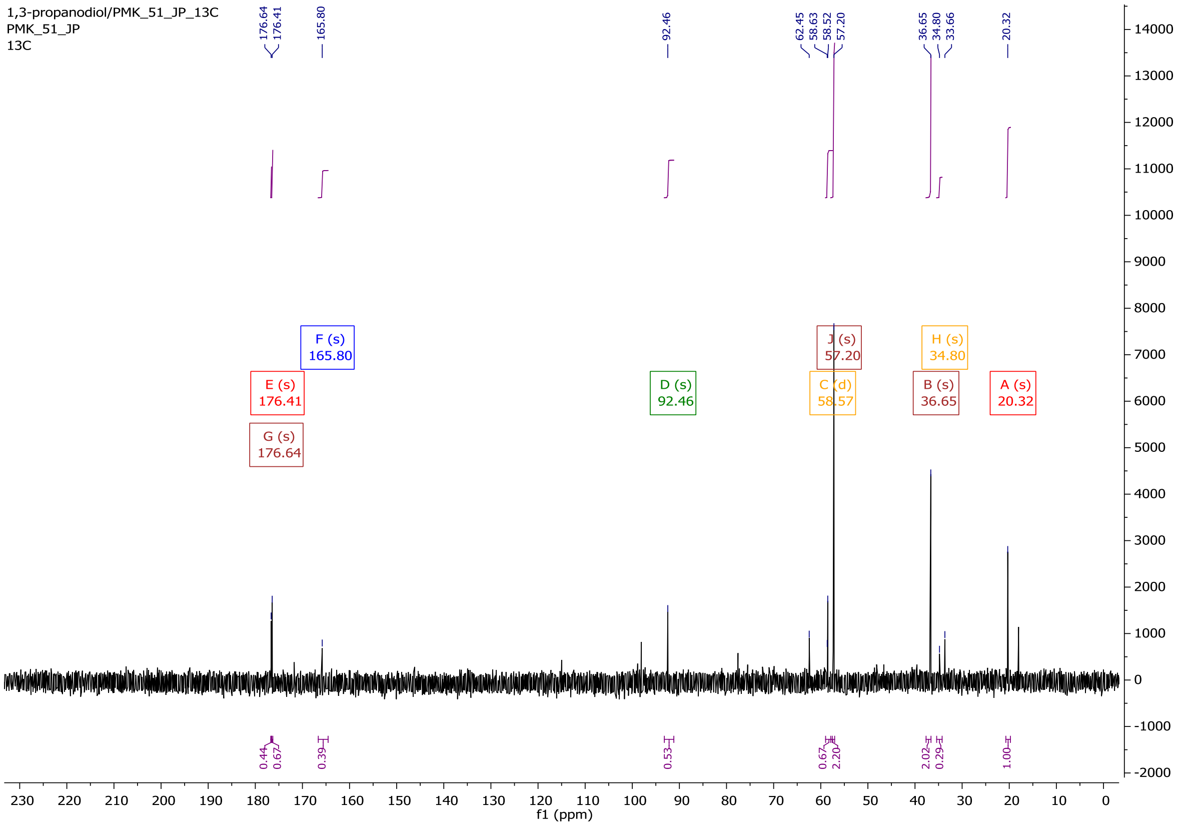
1,3-propanodiol/PMK_51_JP_13C
PMK_51_JP
13C
176.64
176.41
165.80
92.46
62.45
58.63
58.52
57.20
36.65
34.80
33.66
20.32
F (s) 165.80
E (s) 176.41
G (s) 176.64
D (s) 92.46
J (s) 57.20
C (d) 58.57
H (s) 34.80
B (s) 36.65
A (s) 20.32
f1 (ppm)

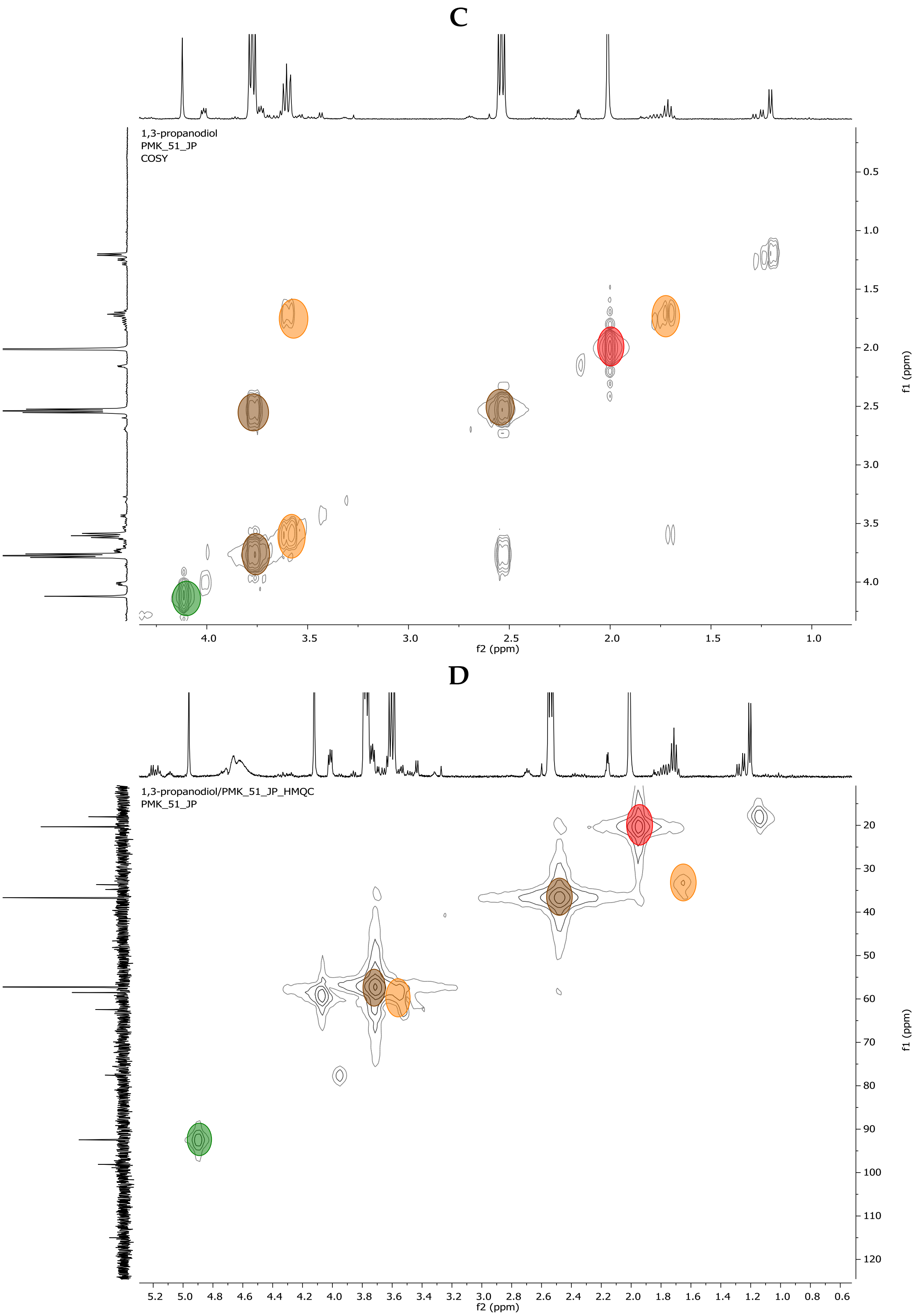

Figure 2. The ^{1}H NMR (**A**), ^{13}C NMR (**B**), COSY (**C**) and HMQC (**D**) spectra of the crude reaction mixture that was required for the catalytic oxidation of 1,3-propanediol in the presence of 0.1% Au/SiO_2 under the reaction conditions as described in Experiment 2.3. Specific signals on the spectra were assigned to the products of the reaction, namely: ● 1,2-propanediol, ● formic acid, ● acetic acid, ● 3-hydroxypropionic acid, ● 1,3,5-trioxane. Other products were not marked on the spectra. Measurements of the samples were taken in D_2O at Bruker 400 MHz.
